# Immunogenic cell death related risk model to delineate ferroptosis pathway and predict immunotherapy response of patients with GBM

**DOI:** 10.3389/fimmu.2022.992855

**Published:** 2022-09-26

**Authors:** Songshan Feng, Xisong Liang, Jing Li, Zeyu Wang, Hao Zhang, Ziyu Dai, Peng Luo, Zaoqu Liu, Jian Zhang, Xiaoxiong Xiao, Quan Cheng

**Affiliations:** ^1^ Department of Neurosurgery, Xiangya Hospital, Central South University, Changsha, China; ^2^ National Clinical Research Center for Geriatric Disorders, Xiangya Hospital, Central South University, Changsha, China; ^3^ Xiangya Cancer Center, Xiangya Hospital, Central South University, Changsha, China; ^4^ Key Laboratory of Molecular Radiation Oncology Hunan Province, Changsha, China; ^5^ Department of Rehabilitation, The Second Xiangya Hospital, Central South University, Changsha, China; ^6^ Department of Oncology, Zhujiang Hospital, Southern Medical University, Guangzhou, China; ^7^ Department of Interventional Radiology, The First Affiliated Hospital of Zhengzhou University, Zhengzhou, China; ^8^ Department of Thoracic Surgery, Xiangya Hospital, Central South University, Changsha, China; ^9^ Department of Clinical Pharmacology, Xiangya Hospital, Central South University, Changsha, China

**Keywords:** immunogenic cell death, risk model, ferroptosis, prognosis, immunotherapy, glioblastoma

## Abstract

Immunogenic cell death (ICD) is a type of cell death that leads to the regulation and activation of the immune response, which is marked by the exposure and delivery of damage‐associated molecular patterns (DAMPs) in the tumor microenvironment. Accumulating evidence has revealed the significance of ICD-related genes in tumor progression and therapeutic response. In this study, we obtained two ICD-related clusters for glioblastoma (GBM) by applying consensus clustering, and further constructed a risk signature on account of the prognostic ICD genes. Based on the risk signature, we found that higher risk scores were associated with worse patient prognosis. Besides, the results illustrated that ferroptosis regulators/markers were highly enriched the high-risk group, and ferroptosis were correlated with cytokine signaling pathway and other immune-related pathways. We also discovered that high-risk scores were correlated to specific immune infiltration patterns and good response to immune checkpoint blockade (ICB) treatment. In conclusion, our study highlights the significance of ICD-related genes as prognostic biomarkers and immune response indicators in GBM. And the risk signature integrating prognostic genes possessed significant potential value to predict the prognosis of patients and the efficacy of ICB treatment.

## Introduction

Immunogenic cell death (ICD) is a form of cell death that leads to the regulation and activation of the immune response, which is characterized by the exposure and release of damage‐associated molecular patterns (DAMPs) in the tumor microenvironment such as calreticulin (CRT), secreted HMGB1and ATP (1, 2). These molecules are signals for immune stimulating effects, including the recruitment and activation of neutrophils, macrophages and other immune cells (3). This feature makes the ICD signature closely related to patients’ prognosis and response to immunotherapy (4). ICD integrates multiple immune-related pathways into a single paradigm, which provides significant advantages as biomarkers (4, 5). There are already studies applying ICD as a marker to predict the survival and immunotherapy response of patients with cancer (4, 6, 7). However, some of the results are contradictory, which may be caused by the differences of specific ICD molecules and the heterogeneity of different tumors (4). Therefore, further research based on the particular ICD molecules categorization and tumor type to identify novel prognostic indicators and therapeutic targets would be beneficial.

Glioblastoma (GBM) the most fatal brain cancer with a median survival time of 12 to 18 months and 5-year survival rate of less than 10% (8, 9). At present, the standard treatment of GBM is the maximum safe surgical resection followed by radiotherapy (RT) plus temozolomide (TMZ) chemotherapy (9, 10). However, due to the invasive growth of GBM in the brain, easy resistance to radiotherapy and chemotherapy, and lack of targeted carcinogenic signal pathway, the traditional comprehensive treatment strategy is difficult to significantly improve the prognosis of GBM patients (11). Therefore, immunotherapy and other emerging therapies have increasingly become the research hotspot of GBM therapy (12, 13). Yet so far, remarkable progress has been made in the treatment of GBM with immune checkpoint blocking (ICB) therapy, which probably resulted from the tumor-related immunosuppressive microenvironment of GBM (14, 15). Besides, no ideal marker can identify patients who will benefit from immunotherapy. Therefore, we conducted this study to discover ICD related prognostic factors and establish a risk model to forecast the prognosis and immunotherapy response of patients with GBM. We also delineated the ferroptosis-related genes (FRGs) and pathways, and predicted response to anti-cancer drugs of GBM patients based on this risk model.

## Materials and methods

### Datasets

For the training set, the RNA-Array data and corresponding clinicopathological information of GBM patients were retrieved from TCGA (http://www.tcga.org/ ). For the validation dataset, the RNA-Seq data were obtained from TCGA. The information about the response of tumor patients to anti-PD-1 checkpoint inhibition therapy was acquired from the Gene Expression Omnibus (GEO) datasets GSE67501 and GSE78220.

### Consensus clustering

Consensus clustering is a clustering algorithm to identify potential clusters with intrinsic heterogeneity. This method indicates the consistency of multiple runs and evaluates the stability of the clusters that was identified during the algorithm process (16). The ConcensusClusterPlus tool in R was applied to conduct consensus clusteriThere are already studies applying ICD as a markerng to obtain particular ICD related clusters for the downstream analysis.

### Construction of the ICD-related risk signature

Significant prognostic ICD genes were selected by univariate regression analysis and a prognostic model was constructed by ridge regression further (17). The risk score was calculated by the exact coefficient value of each associated gene. Then both the training and validation cohort was divided into two groups according to the risk score for further study.

### Survival analysis

Kaplan Meier curves and log rank test were applied to compare the overall survival (OS), progression free survival (PFI), and disease-specific survival (DSS) of patients in different clusters or risk groups. ROC curve was utilized to estimate the predictive performance of different classification methods in various aspects, including 1 -, 3 -, and 5-year survival, GBM subtypes, and IDH status.

### Differentially expressed genes (DEGs) identification and functional enrichment analysis

The DEGs between different risk groups were identified by utilizing the R package “Limma”. Gene Ontology (GO) and Kyoto Encyclopedia of Genes and Genomes (KEGG) analyses of the aberrantly expressed genes were determined by the gene set variation analysis (GSVA), which compared the variation levels of gene sets between groups (18). We also conducted the gene set enrichment analysis (GSEA) to calculate the enrichment score of the target gene sets including GO and KEGG. GSEA is based on genomewide expression profiles from two classes of samples, and ranks genes according to the correlation between gene expression and the class distinction (19).

### ESTIMATE score analysis

ESTIMATE (Estimation of Stromal and Immune cells in MAlignant Tumor tissues using Expression data) is an implement to estimate tumor purity and the presence of infiltrating stromal cells/immune cells in tumor tissues utilizing gene expression according to the enrichment analysis of single sample gene set (20).

### Immune infiltration analysis

The immune infiltration heterogeneity between groups was estimated by the R package integrating six state-of-the-art algorithms, which include TIMER (21), MCP-counter (22), EPIC (23), xCell (24), quanTIseq (25) and CIBERSORT (26). Each of them possesses unique analysis scenarios and capabilities. The results were displayed as heatmap and quantitative box plots.

### TIDE score analysis

Tumor immune dysfunction and exclusion (TIDE) analysis was performed to evaluate the potential response to ICB treatment. TIDE (http://tide.dfci.harvard.edu/) assessed two different indexes of tumor immune escape, including dysfunction of cytotoxic T lymphocytes (CTLs) and rejection of CTLs by immunosuppressive molecules (27, 28). Patients with a higher TIDE score indicates a higher probability of tumor immune escape and thus lower ICB therapy response rate.

### GDSC database

The Genomics of Drug Sensitivity in Cancer (GDSC) database was utilized to locate the drugs with the specific target potential as anti-cancer candidates based on the correlation between the risk score and drug response AUC. GDSC database (www.cancerRxgene.org) collects numerous cell drug sensitivity information and drug disturbance-based cell expression signature, which provides specific information for integrating a large number of drug sensitivity and genomic data to promote the discovery of novel therapeutic biomarkers for cancer treatment (29).

### Statistical analysis

The survival differences between different clusters or risk groups were explored by the log-rank test. According to the median risk score of all patients, patients with higher risk scores are divided into a high-risk group while the others were assigned into the low-risk group. The comparison of normally-distributed or non-normally distributed parameters between two groups were tested by Wilcoxon rank testing. The Pearson correlation was applied to evaluate the linear relationship between risk score and hallmark pathways. P value < 0.05 was considered to be statistically significant.

## Results

### Construction and validation of ICD-related risk signature

**Figure 1:**
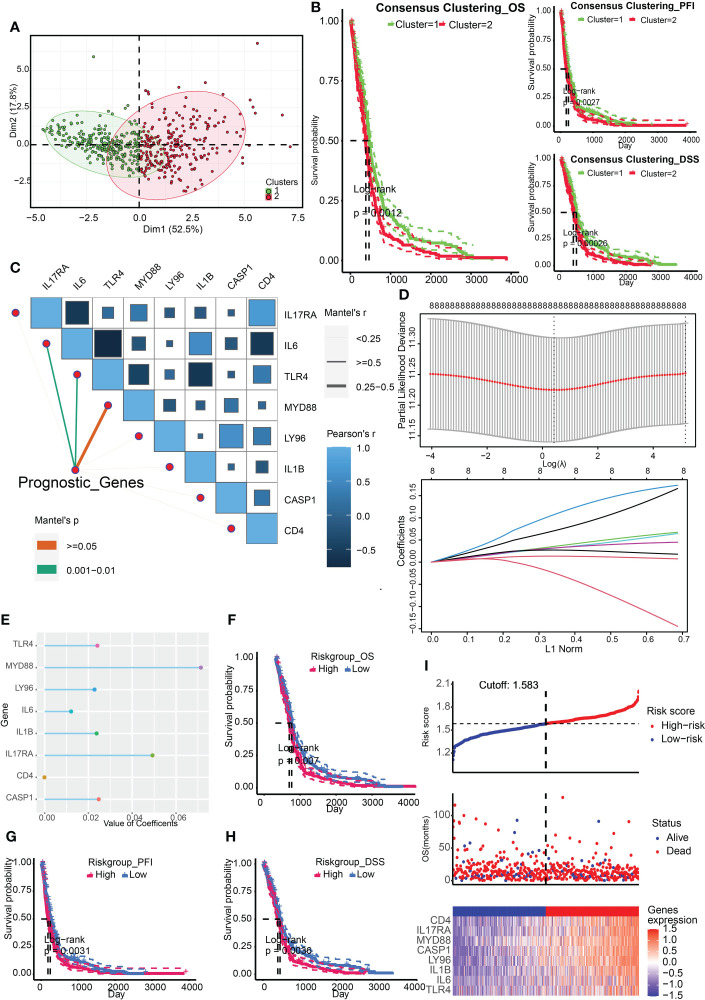
Construction of ICD related clusters and risk signature. **(A)** Two ICD related clusters were obtained from the TCGA Array dataset by applying consensus clustering. **(B)** Survival analysis indicated that cluster 2 was associated with a dismal prognosis from the perspective of OS, PFI, and DSS. **(C)** Univariate regression analysis identified 8 significant prognostic ICD genes including IL17RA, IL6, TLR4, MYD88, LY96, IL1B, CASP1, and CD4. **(D)** The eight genes were tested for the prediction model by ridge regression. **(E)** The correlation coefficients of the eight genes were displayed. **(F-H)** Survival analysis indicated that a higher risk score was correlated to worse OS **(F)**, PFI **(G)**, and DSS **(H)**. **(I)** Risk scores distribution, patient survival status, and gene expression heatmap were displayed. P < 0.05, statistically significant.

The ICD associated genes were summarized by Abhishek D Garg et al. in the previous study (4). We firstly obtained two ICD related clusters from the TCGA Array dataset by applying consensus clustering (**Figure 1A**). The ICD gene level was higher in cluster 2. Survival analysis indicated that cluster 2 was associated with a dismal prognosis from the perspective of OS, PFI, and DSS (**Figure 1B**). After that, we identified 8 significant prognostic ICD genes through univariate regression analysis including IL17RA, IL6, TLR4, MYD88, LY96, IL1B, CASP1, and CD4 (**Figure 1C**), which were tested for the prediction model by ridge regression further (**Figure 1D**). The risk score is equal to the sum of each significant gene multiplied by the correlation coefficient (**Figure 1E**). The algorithm was as below: Risk score= 0.0493* IL17RA +0.0121*IL6+0.0241*TLR4+0.0714*MYD88+0.0228*LY96+0.0237*IL1B+0.0247*CASP1- 8.8893e-05*CD4. We also found that higher risk scores predicted worse outcomes (**Figures 1F-H**). The patient cohort was divided into two groups according to the median risk score (1.583), and the survival risk heatmap were displayed in **Figure 1I**.

**Figure 2:**
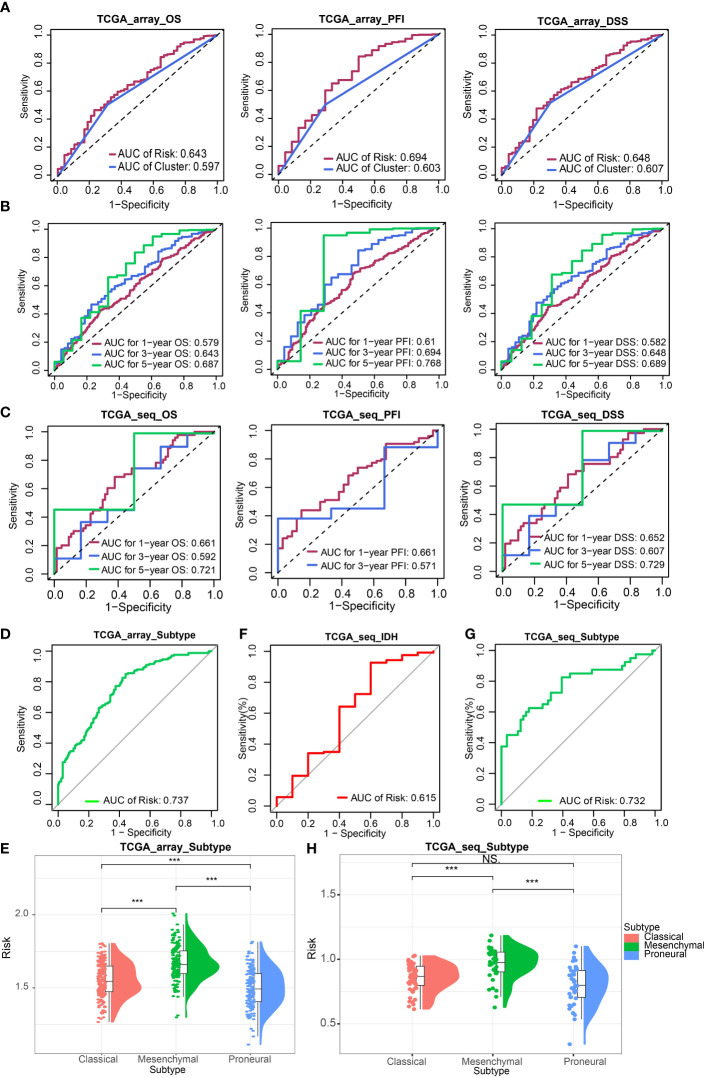
Validation of ICD-related risk signature. **(A)** AUC of survival prediction indicated the prediction efficacy of risk signature was better than clusters. **(B, C)** AUC for 1 -, 3 -, and 5-year survival prediction in TCGA Array dataset **(B)** and TCGA Seq dataset **(C)**. **(D)** AUC for GBM subtypes prediction in TCGA Array dataset. **(E)** The risk scores of different GBM subtypes (classical, mesenchymal, proneural) in the TCGA Array dataset. **(F)** AUC for IDH status prediction in TCGA Seq dataset. **(G)** AUC for GBM subtypes prediction in TCGA Seq dataset. **(H)** The risk scores of different GBM subtypes in the TCGA Seq dataset. NS, non-significant. ***P < 0.001.

Next, we evaluated the effectiveness of the risk signature by ROC curve and AUC. The prognostic prediction capability of risk signature and clusters were determined. The results of AUC in the TCGA Array dataset suggested that the prediction efficacy of risk signature was better than clusters (**Figure 2A**). Then We calculated the AUC for estimating the prediction accuracy of survival time of 1 year, 3 years, and 5 years, respectively in the TCGA Array dataset (**Figure 2B**) and TCGA Seq dataset (**Figure 2C**) based on the risk signature. The AUC according to GBM subtypes (**Figures 2D, G**) and the status of IDH (**Figure 2F**) prediction were also obtained.

The risk scores of different GBM subtypes (classical, mesenchymal, proneural) were compared in both the TCGA array (**Figure 2E**) and the TCGA Seq data sets (**Figure 2H**). The results both revealed that the mesenchymal subtype possessed the highest risk score.

### The ICD groups differ in cancer hallmark, immune, and cell death-related pathways

**Figure 3:**
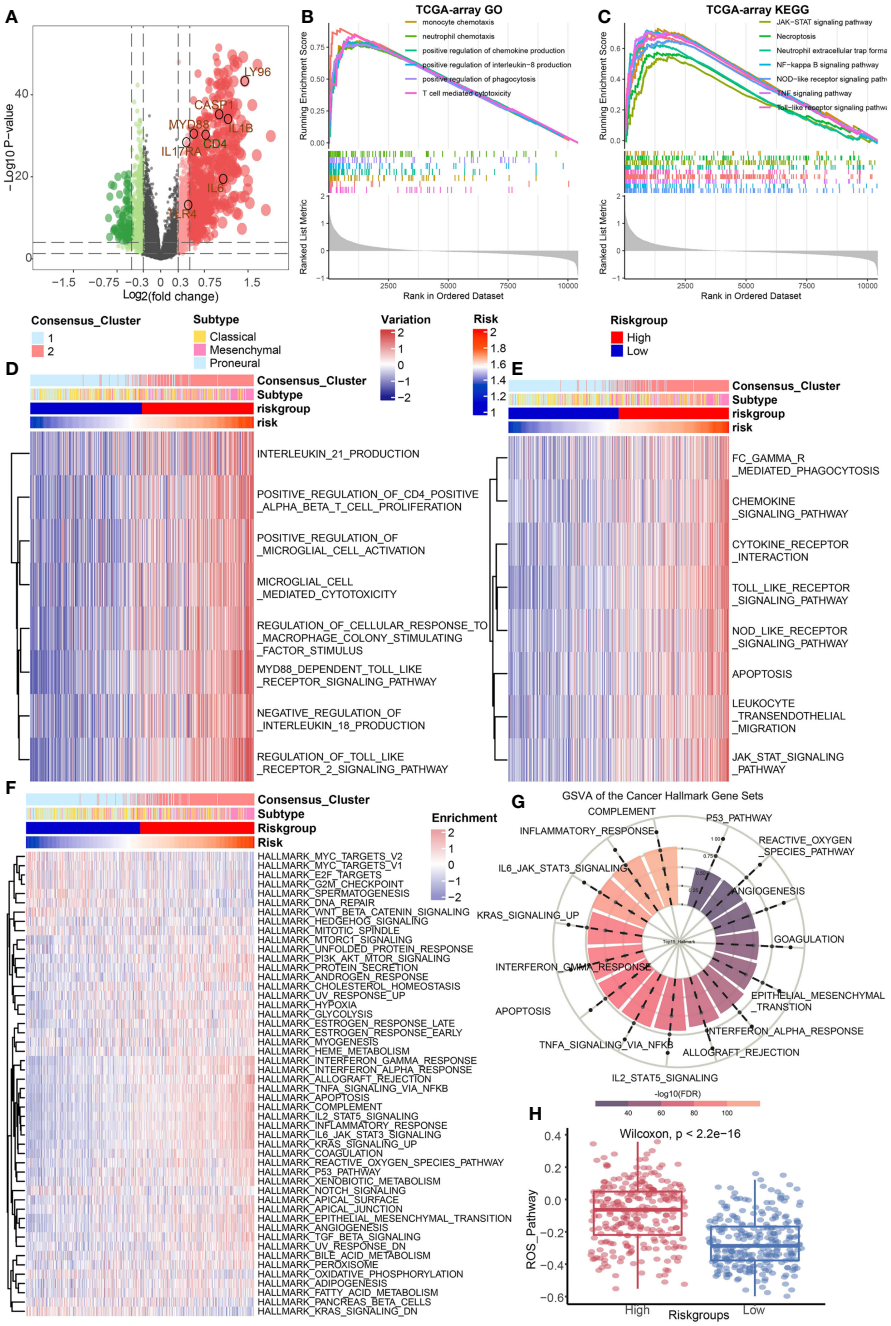
Identification of DEGs and functional enrichment analysis. **(A)** The differentially expressed genes between high and low risk group were identified based on the TCGA Array dataset. **(B, C)** GSEA results of GO terms **(B)** and KEGG pathways **(C)** based on the differentially expressed gene sets in the TCGA Array dataset. **(D, E)** GSVA results of GO terms **(D)** and KEGG pathways **(E)** based on the differentially expressed gene sets in the TCGA Array dataset. **(F, G)** ssGSEA results of the 50 “hallmark” gene sets from the Molecular Signature Database. **(H)** ssGSEA results suggested the ROS pathway was enriched in the high-risk group. P < 0.05, statistically significant.

The DEGs between the high-risk group and the low-risk group in TCGA Array dataset were screened out (**Figure 3A**). GSEA was conducted to illustrate the enriched GO terms (**Figure 3B**) or KEGG pathways (**Figure 3C**) according to these DEGs. The results revealed that the enriched GO terms were mostly related to immune regulation pathways such as monocyte chemotaxis, neutrophil chemotaxis, positive regulation of chemokine production, and so on. The GSEA results based on the TCGA Seq dataset were basically consistent (**Supplementary Figures 1A, B**). GSVA results of GO terms (**Figure 3D**) and KEGG pathways (**Figure 3E**) also suggested that immune regulatory pathways such as chemokine signaling pathways were enriched in the high-risk group. The similar GSVA results based on the TCGA Seq dataset were displayed in **Supplementary Figures 1C, D**. Additionally, the single sample GSEA (ssGSEA) of the hallmark gene sets originated from MsigDB were performed (30). The results demonstrated that the complement pathway, inflammatory response, and IL6-JAK-STAT3 hallmark pathways were enriched in the high-risk group (**Figures 3F, G**). These functional enrichment results indicated that immune related signals and pathways were active in the high-risk group. The reactive oxygen species (ROS) pathway was also enriched in the high-risk group, implying a high correlation between cell death and tumor immunity (**Figure 3H**).

### Relationship between risk signature and different cell death modes

**Figure 4:**
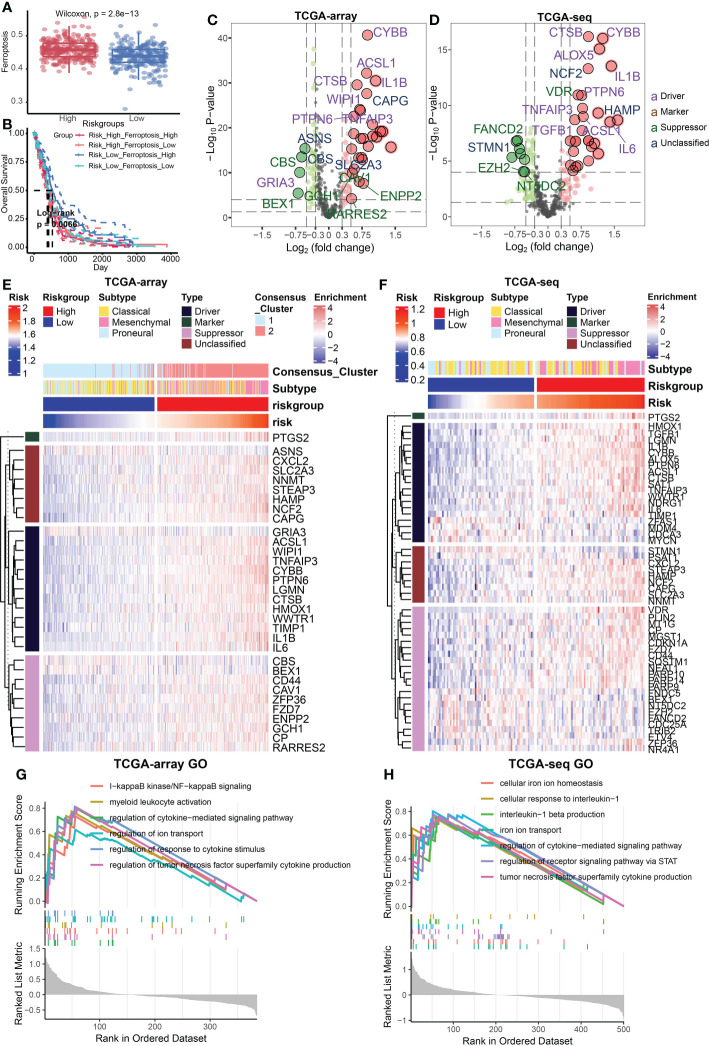
Relationship between risk signature and different cell death modes. **(A)** Ferroptosis regulators/markers were highly enriched in high-risk group based on the TCGA Array dataset. **(B)** Survival analysis revealed that the group with low-risk and high levels of ferroptosis possessed the best prognosis. **(C, D)** The volcano plots showed differentially expressed FRGs in the TCGA Array dataset **(C)** and TCGA Seq dataset **(D)**. **(E, F)** The heatmap displayed differentially expressed FRGs in the TCGA Array dataset **(E)** and TCGA Seq dataset **(F)**. **(G, H)** ssGSEA results of GO terms in TCGA Array dataset **(G)** and TCGA Seq dataset **(H)**. P < 0.05, statistically significant.

**Figure 5:**
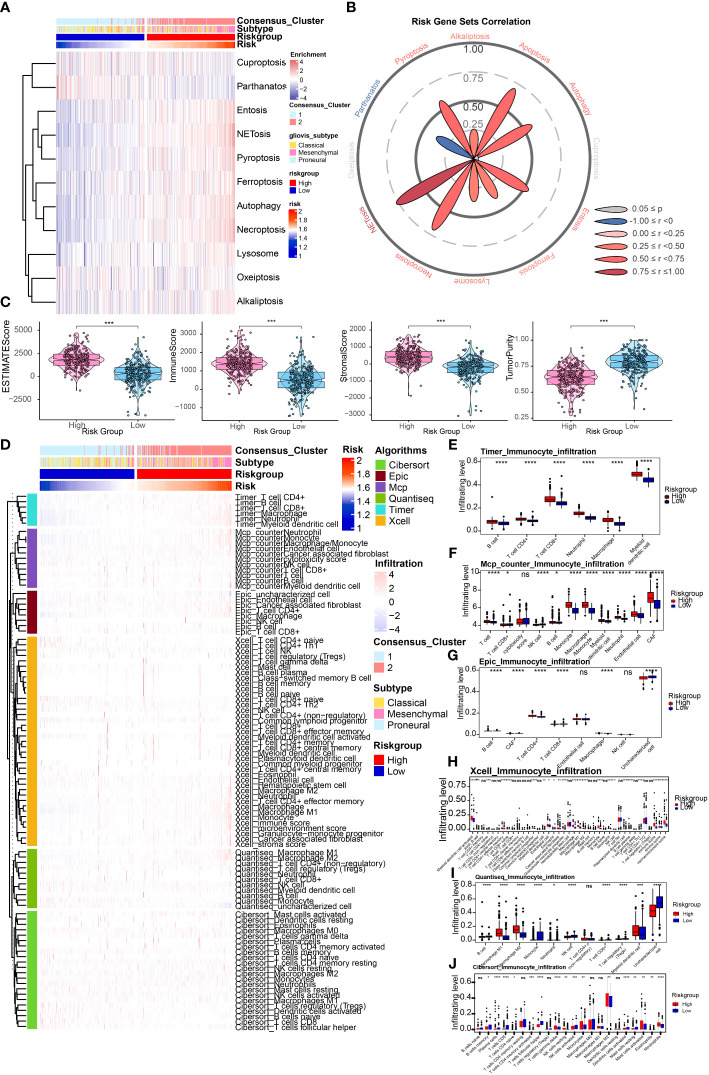
Depiction of tumor immune microenvironment on account of risk signature. **(A)** ssGSEA results of different cell death modes in TCGA Array dataset. **(B)** Correlation analysis of risk score and different cell death modes. **(C)** ESTIMATE score to predict tumor purity and the presence of infiltrating stromal cells/immune cells in tumor tissues based on the TCGA Array dataset. **(D)** Heatmap illustrated immune infiltration by performing the R package which integrates TIMER, MCP-counter, EPIC, xCell, quanTIseq, and CIBERSORT in the TCGA Array dataset. **(E-J)** Box plots displayed immune infiltration differences between high and low risk group by applying TIMER **(E)**, MCP-counter **(F)**, EPIC **(G)**, xCell **(H)**, quanTIseq **(I)**, and CIBERSORT **(J)**. NS, non-significant. *P < 0.05, **P < 0.01, ***P < 0.001, ****P < 0.0001.

Ferroptosis is an important programmed cell death mode. We next attempted to delineate the relationship between risk signature and regulators/markers of ferroptosis (31). Firstly, we found that ferroptosis regulators/markers was highly enriched in the high-risk group in the TCGA Array dataset (**Figure 4A**) and TCGA Seq dataset (**Supplementary Figure 2A**). We further divided patients into 4 groups on account of risk score and ferroptosis pathway level. Survival analysis revealed that the group with low-risk and high level of ferroptosis possessed the best prognosis (**Figure 4B**). However, no statistically significant difference in survival rate was observed in the TCGA seq dataset (**Supplementary Figure 2B**). Then we analyzed the differentially expressed FRGs in the TCGA Array dataset (**Figure 4C**) and TCGA Seq dataset (**Figure 4D**). The results suggested that most of the ferroptosis driver genes were upregulated in the high-risk group. The heatmap displaying DEGs illustrated similar results (**Figures 4E, F**). Additionally, we performed ssGSEA of GO terms in the TCGA Array dataset (**Figure 4G**) and TCGA Seq dataset (**Figure 4H**), and found that ion transport, regulation of cytokine signaling pathway and other immune related pathways were highly enriched in the high-risk group. Besides, we compared the level of cell-death-related gene sets between the risk groups and analyzed their correlation with risk score in TCGA Array (**Figures 5A, B**) and TCGA Seq dataset (**Supplementary Figure 2C**). The results showed that entosis, netosis, pyroptosis, autophagy, and necroptosis were enriched in high-risk group (**Figures 5A, B**).

### Depiction of tumor immune microenvironment on account of risk signature

**Figure 6:**
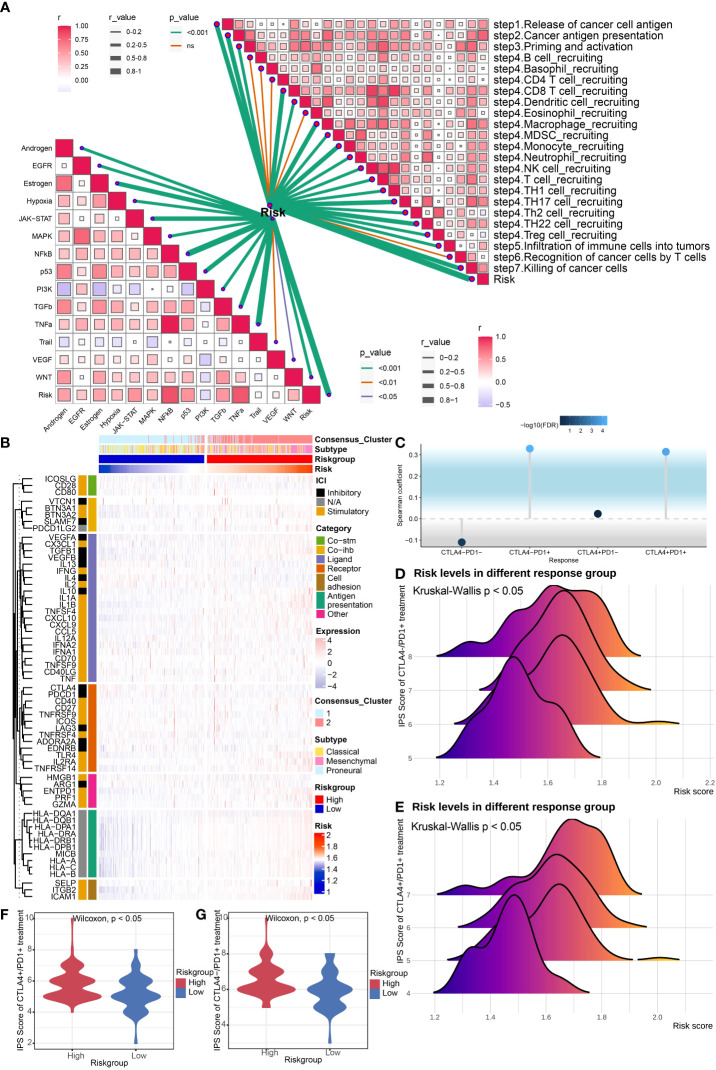
Evaluation of potential response to ICB treatment. **(A)** Correlation analysis of risk score and anti-cancer immunity cycle and 11 cancer related pathways. **(B)** Heatmap revealed immune associated gene expression levels in the TCGA Array dataset within the two risk groups. **(C)** The lollipop plot showed the correlation between risk core and ICB treatment response. **(D, E)** The ridgeline plots displayed the risk levels in the CTLA4-/PD1+ treatment group **(D)** and CTLA4+/PD1+ treatment group **(E)**. **(F, G)** The violin plots revealed IPS scores of high/low risk group in the CTLA4+/PD1+ treatment group **(F)** and CTLA4-/PD1+ treatment group **(G)**. P < 0.05, statistically significant.

We first utilized the ESTIMATE score to estimate the immune infiltration in tumor tissues. The results revealed that the ESTIMATE score, Immune score, and Stromal score were higher in high-risk group, and tumor purity score was lower, which indicated higher infiltrating stromal cells/immune cells in high-risk group (**Figure 5C**). A similar tendency was identified utilizing the TCGA Seq dataset (**Supplementary Figure 3A**). Then we conducted the immune infiltration estimations by performing the R package which integrates TIMER, MCP-counter, EPIC, xCell, quanTIseq, and CIBERSORT in the TCGA Array dataset (**Figures 5D-J**) and TCGA Seq dataset (**Supplementary Figure 3B**). Results of TIMER illustrated a higher infiltrating level of immune cells in the high-risk group. All the six methods indicated higher macrophage infiltrating levels in tumors, especially M2 macrophages, which suggested higher risk score may be correlated with the inhibitory immune microenvironment. Moreover, results of MCP-counter, EPIC, xCell, quanTIseq, and CIBERSORT revealed that infiltrating level of CD8+ T cell was lower in high-risk group, suggesting weaker immune defense against tumor in high-risk group. We also estimated the risk score's correlation with anti-cancer immunity cycle (32) or 11 cancer related pathways (33), which indicated high relevance of risk score with them (**Figure 6A**). The results suggested that the risk score was more relevant to macrophage recruiting or monocyte recruiting regarding the anti-cancer immunity cycle, and more relevant to NFκB or TNFα pathways as regarding 11 cancer related pathways.

### Evaluation of potential response to ICB treatment

**Figure 7:**
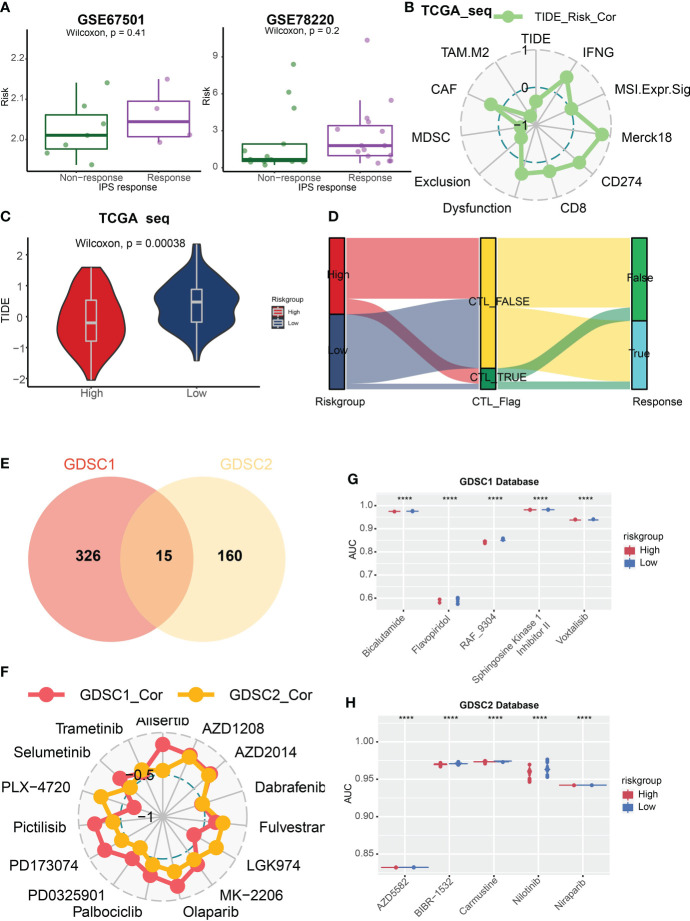
TIDE score and GDSC database analysis. **(A)** The box plots revealed the risk score of the response or non-response group in datasets GSE67501 and GSE78220. **(B)** Correlation analysis of risk score and TIDE associated scores. **(C)** The violin plot showed that the TIDE score was lower in high-risk group. **(D)** The Sankey diagram illustrated the prediction of ICB treatment response with respect to risk group. **(E)** 341 and 175 kinds of potential drug were identified in GDSC1 and GDSC2 databases respectively, and 15 kinds of them were overlapped whose AUC values were negatively correlated with risk score. **(F)** The relationship between these 15 drugs and risk score. **(G, H)** The box plots revealed five drugs with the greatest negative correlation with risk scores in the GDSC1 **(G)** and GDSC2 database **(H)** respectively. P < 0.05, statistically significant. ****P < 0.0001.

We evaluated immune associated gene expression levels in the TCGA Array dataset within the two risk groups, and found that human leukocyte antigens (HLAs), the key molecules mediating antigen presentation in cancer immunotherapy, were upregulated in the high-risk group (**Figure 6B**). The gene expression levels in TCGA Seq dataset displayed the same result (**Supplementary Figure 3C**). Then we analyzed the immune positive score (IPS) and its relationship with risk signature by applying the IPS data downloaded from TCIA (The cancer Immunome Atlas, https://tcia.at/home) (34). Notably, the correlation between IPS and risk signature in GBM patients was established (**Figures 6C-G**). The IPS score increased in the high-risk group, including both CTLA4-/PD1+ treatment and CTLA4+/PD1+ treatment subgroup, which indicated that the high-risk group patients may benefit from the anti-PD1 treatment. We also predicted the response of tumor patients to anti-PD-1 checkpoint inhibition therapy in datasets GSE67501 and GSE78220, and found that the risk score was higher in the response group of both datasets. Yet no statistically significant difference was found (**Figure 7A**). Next, we utilized the TIDE score to predict the immune escape targeted treatment response. The results exhibited that the TIDE score was lower in high-risk group, indicating lower probability of tumor immune escape and thus a higher response rate of ICB therapy in the high-risk group (**Figures 7B, C**). The risk score's correlation with cytotoxic T lymphocyte level and therapeutic responses was displayed in **Figure 7D**.

### Potential effective anti-cancer drugs analysis

Then, we applied the GDSC1 and GDSC2 databases to assess potential effective anti-cancer drugs based on risk scores and the AUC values of drugs. We identified 341 and 175 kinds of potential drugs in GDSC1 and GDSC2 databases respectively, and 15 kinds of them were overlapped whose AUC values were negatively correlated with risk scores (**Figure 7E**). Then we analyzed the relationship between these 15 drugs and risk scores (**Figure 7F**). Next, we discovered five drugs with the greatest negative correlation with risk scores in the GDSC1 or GDSC2 databases respectively. We found that bicalutamide, flavopiridol, RAF-9304, sphingosine kinase 1 inhibitor II` and voxtalisib exhibited lower AUC in the high-risk group based on the GDSC1 database (**Figure 7G**), while AZD5582, BIBR1532, carmustine, nilotinib, and niraparib were most effective potential drugs identified in the GDSC2 database (**Figure 7H**).

### ICD biomarker MYD88 was associated with patient prognosis, cell death and IPS score

At last, we performed the analysis about association between ICD biomarker MYD88 and cancer properties to verify the role of this ICD related risk signature we constructed. We firstly applied GEPIA2 (http://gepia2.cancer-pku.cn/#index) (35) to obtain the expression profile of MYD88, and we found that MYD88 was upregulated in GBM tissues (**Supplementary Figure 4A**). Survival analysis indicated that high MYD88 was associated with a dismal prognosis from the perspective of OS, DSS, and PFI **(** **Supplementary Figure 4B** **)**. We also found that MYD88 expression was the highest in the Mesenchymal subtype **(** **Supplementary Figure 4C** **)**, and it associated with many kinds of cell death pathways, and negatively correlated to PD1 expression (**Supplementary Figures 4D, 4E**) Finally, the correlation between IPS score and MYD88 expression in GBM patients was established **(** **Supplementary Figures 4F** **)**. There was an increase in IPS scores in high-MYD88 patients, including both CTLA4-/PD1+ treatment and CTLA4+/PD1+ treatment subgroup.

## Discussion

**Figure 8:**
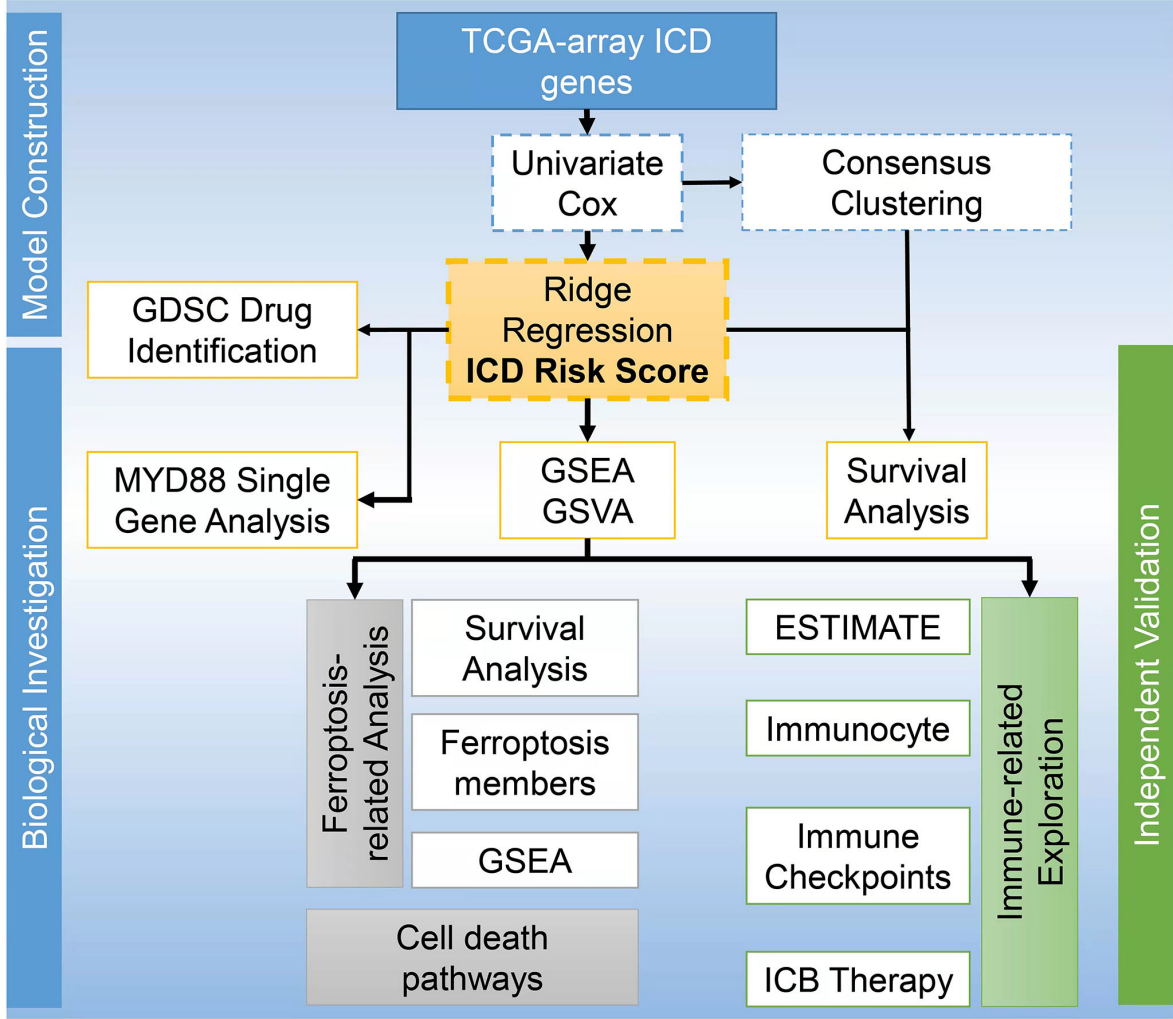
Workflow to present the study design.

ICD, a novel concept linking dying cancer cells with the immune system, is a form of cell death that leads to the regulation and activation of the immune response (36). The concomitant ROS production and endoplasmic reticulum stress within the ICD process activate the exposure and release of DAMPs in the tumor microenvironment, which is able to potentiate immune response (37). Currently, there is no study reporting the prognosis and immunotherapy of patients with GBM based on ICD gene signatures. ICD signature has been discovered to be associated with improved prognosis of lung, breast, and ovarian cancer patients (4). By analyzing these genes, we obtained two ICD related clusters from TCGA Array dataset by applying consensus clustering. The Cluster 2 with higher level of ICD gene expression was associated with a dismal prognosis. Moreover, we built a ICD model based on the 8 ICD genes and grouped the patients according to their risk score. We also found that higher risk scores were associated with worse outcomes. The ROC curves and AUC suggested good predictive efficacy of this risk signature. We have drawn a workflow to present the study design displayed as **Figure 8**.

The ability of ICD to elicit adaptive immunity provides the potential of ICD-eliciting therapy as a partner for immunotherapy, such as chemotherapy and radiotherapy (38). Radiotherapy coordinated with CTLA4 blockade to induce T cell's anti-cancer activity in chemotherapy-resistant lung cancer (39). Nearly half the patients presented object response or disease control, probably due to the mutated immunogen exposure mediated by radiation. Breast carcinoma is one of the most common tumors for the use of ICD-inducing chemotherapy in combination with immunotherapy (40). The current clinical studies expect to apply immune therapy, especially ICB, to achieve optimal therapeutic effects with the use of ICD inducing therapies which can elicit maximal immunostimulation (38).

We identified 8 significant prognostic ICD genes including IL17RA, IL6, TLR4, MYD88, LY96, IL1B, CASP1, and CD4. GBM-derived IL6 was reported to inhibit antitumor immune response against GBM. Meanwhile, inhibition of glioma IL6 in vivo prolonged mouse survival time (41). Other studies revealed that the expression of TLR4 was significantly higher in GBM than in grade III anaplastic astrocytoma, and was correlated with poor patient prognosis (42, 43). And decreased TLR4 expression could inhibit GBM invasiveness, promote apoptosis and improve survival (44). As regarding MYD88, it was found upregulated in gliomas compared to normal tissues and correlated with unfavorable prognosis. Moreover, the authors discovered that MYD88 expression was higher in IDH1 wild types glioma and positively associated with M2 macrophage infiltration (45). The previous relevant studies indicated that our risk signature integrating these genes possessed potential value for prognosis prediction.

Ferroptosis is a novel programmed cell death mode first proposed by Dixon et al. (46). The mechanism of ferroptosis has not been thoroughly elucidated. Its main characteristics include the toxicity of intracellular iron accumulation, lipid peroxidation, and the inhibition of SLC7A11-GSH-GPX4 antioxidant axis (47). Increasing studies have indicated ferroptosis' effects on tumorigenesis, tumor progression, and therapeutic response (47, 48). Ferroptosis has been noticed to release DAMPs or lipid metabolites to regulate immunity, confirming its ICD-like function (49). The released DAMPs promoted dendritic cells and cytotoxic T cell mediated immunity (50). Xiang Luo et al. reported a novel phagocytosis mechanism that oxidized phosphatidylethanolamine on the ferroptotic cell surface increased the efficiency of phagocytosis of ferroptotic cells by macrophage (51). Iuliia Efimova et al. reported that vaccination with early ferroptotic cancer cells induces efficient antitumor immunity by triggering ferroptosis-dependent ICD in preclinical models (52). On the other hand, the cellular immune response can also affect the ferroptosis process. Weimin Wang et al. reported that CD8+ T cell derived IFNγ promoted cancer peroxidation and ferroptosis by downregulating SLC3A2 and SLC7A11 expression. (53). Our results suggested that ferroptosis regulators/markers were highly enriched in the high-risk group, and ferroptosis were correlated with cytokine signaling pathway and other immune related pathways, suggesting its potential function in immune regulation.

Tianqi Liu and his colleagues reported in a newly published study that ferroptosis was the major kind of programmed cell death in glioma, and elevated ferroptosis could induce activation of immune cells but inhibit antitumor cytotoxic killing resulted from the infiltration of tumor-associated macrophages (54). The results were partly consistent with our findings that higher risk scores were associated with higher macrophage infiltrating levels and lower infiltrating level of CD8+ T cell. The authors also discovered a novel synergistic immunotherapeutic therapy combining ferroptosis inhibition with ICB treatment through GBM murine models. This is superior to our study since our study lacks *in vivo* and *in vitro* experimental validation.

ICB treatment has been applied as a first line therapy for advanced melanoma, non-small cell lung cancer, clear cell renal cell carcinoma, and solid tumors containing DNA mismatch repair defects or microsatellite instability, bringing long-term disease remission for some patients with advanced malignant tumors (55). However, no significant progress in ICB therapy has been made in GBM. A phase 3 clinical trial of PD-1 blocker nivolumab and bevacizumab in the treatment of recurrent GBM suggested that compared with bevacizumab, nivolumab did not improve the overall survival rate of patients (14). The tumor related immunosuppressive microenvironment of GBM may contribute to the poor response to ICB treatment (11, 15). Besides, no ideal marker can distinguish the patients who may benefit from immunotherapy. Víctor A Arrieta et al. reported in a recent study that the activation of ERK1/2 in recurrent GBM could predict the therapeutic response to PD-1 blockade and was related to unique myeloid cell phenotype (56). Daniel J McGrail et al. reported that inducing ICD with MLN4924 combined with PD1 inhibition was synergistic and could significantly improve treatment efficacy (57). Some clinical trials have now confirmed that pretreatment with drugs that induce ICD can sensitize ICB treatment against PD-1 and PD-L1 interactions (58). We found that HLAs, the key molecules mediating antigen presentation in cancer immunotherapy, were upregulated in the high-risk group. The increase in IPS score in the high-risk group also revealed that these patients were more likely to benefit from ICB treatment. Furthermore, the TIDE score was lower in the high-risk group, indicating a higher response rate of ICB treatment. These results revealed the risk score's potential for indicating ICB treatment response.

In conclusion, our study highlights the significance of ICD related genes as prognostic biomarkers and immune response indicators in GBM. And the risk signature integrating prognostic genes possessed significant potential value to predict glioblastoma patients' prognosis and ICB treatment efficacy.

## Data availability statement

The datasets presented in this study can be found in online repositories. The names of the repository/repositories and accession number(s) can be found in the article/**Supplementary Material**.

## Author contributions

SF, XX, and QC made substantial contributions to the design of this study. XL, JL, ZL, and ZW carried out the analysis and interpreted the data. SF, XL, and XX made contributions to the drafting of the manuscript. HZ, ZD, PL, and JZ made contributions to the review of previous literature. SF, XX, XL, and QC contributed substantially to the revision of the manuscript. All authors contributed to the article and approved the submitted version.

## Funding

This work was supported by the National Natural Science Foundation of China (NO.82102848, NO.82073893, NO.81703622); Hunan Provincial Natural Science Foundation of China (No.2020JJ8111, NO.2022JJ20095, 2022JJ40830); Hunan Provincial Health Committee Foundation of China (NO.202204044869); Natural Science Foundation General Program of Changsha City (kq2014290); and Xiangya Hospital Central South University postdoctoral foundation.

## Acknowledgments

All authors thank TCGA and GEO database for providing the wothy patient cohort data for research, and we also thank Central South University for providing the High Performance Computing Center.

## Conflict of interest

The authors declare that the research was conducted in the absence of any commercial or financial relationships that could be construed as a potential conflict of interest.

## Publisher’s note

All claims expressed in this article are solely those of the authors and do not necessarily represent those of their affiliated organizations, or those of the publisher, the editors and the reviewers. Any product that may be evaluated in this article, or claim that may be made by its manufacturer, is not guaranteed or endorsed by the publisher.

## Supplementary material

Supplementary Figure 1 Functional enrichment analysis in TCGA Seq dataset. **(A, B)** GSEA results of GO terms **(A)** and KEGG pathways **(B)** based on the differentially expressed gene sets in the TCGA Seq dataset. **(C, D)** GSVA results of GO terms **(C)** and KEGG pathways **(D)** based on the differentially expressed gene sets in the TCGA Seq dataset.

Supplementary Figure 2 Relationship between risk signature and different cell death modes in TCGA Seq dataset. **(A)** Ferroptosis regulators/markers were highly enriched in high-risk group based on the TCGA Seq dataset. **(B)** Survival analysis revealed no significance within the four groups on account of risk score and ferroptosis pathway level in the TCGA Seq dataset. **(C)** ssGSEA results of different cell death modes in TCGA Seq dataset. P < 0.05, statistically significant.

Supplementary Figure 3 Depiction of tumor immune microenvironment and immune associated genes in TCGA Seq dataset. **(A)** Calculation of the ESTIMATE score, ImmuneScore, StromalScore, and TumorPurity of the TCCA-seq samples. **(B)** Heatmap illustrated immune infiltration by performing the R package which integrates TIMER, MCP-counter, EPIC, xCell, quanTIseq, and CIBERSORT in the TCGA Seq dataset. **(C)** Heatmap revealed immune associated gene expression levels in the TCGA Seq dataset within the two risk groups. P < 0.05, statistically significant.

Supplementary Figure 4 ICD biomarker MYD88 was associated with clinical malignancy, cell death pathways, and IPS score. **(A)** Expression differences analysis of MYD88 in GBM and control tissue based on GEPIA2. **(B)** Survival analysis indicated that high MYD88 was associated with a dismal prognosis. **(C)** MYD88 expression differences in different GBM subtypes. **(D)** The correlation analysis indicated that MYD88 expression was associated with different kinds of cell death modes. **(E)** The correlation analysis revealed that MYD88 expression was negatively correlated with tumor PD-1 expression. **(F)** The MYD88 expression levels in GBM patients with different IPS scores.
