# Supplementary figures and images for: Immunogenic cell death related risk model to delineate ferroptosis pathway and predict immunotherapy response of patients with GBM
Source: Front Immunol. 2022 Sep 26;13:992855. doi: 10.3389/fimmu.2022.992855 (PMC9554879; doi:10.3389/fimmu.2022.992855)

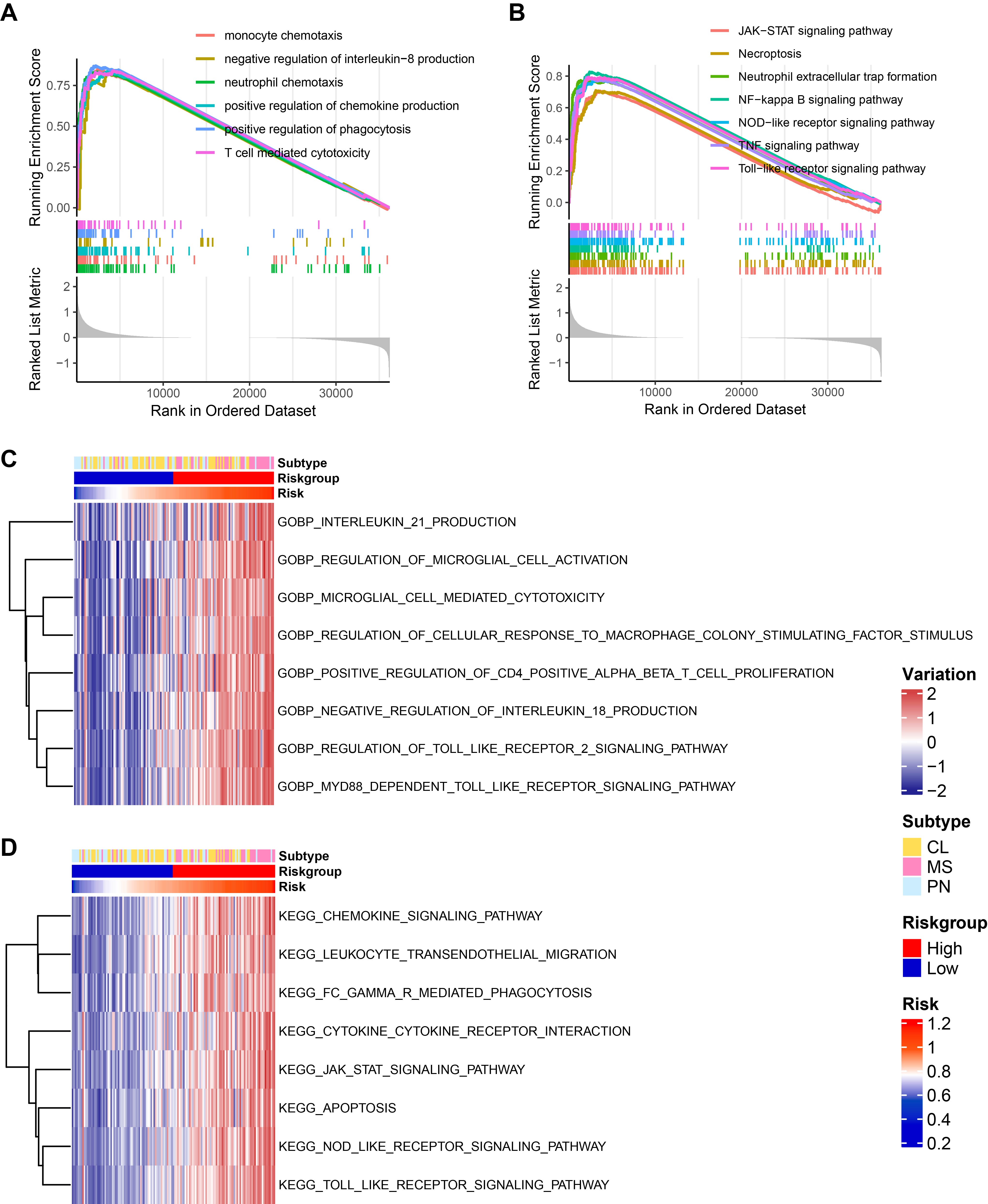

Supplement: Supplementary Figure 1 — Functional enrichment analysis in TCGA Seq dataset. (A, B) GSEA results of GO terms (A) and KEGG pathways (B) based on the differentially expressed gene sets in the TCGA Seq dataset. (C, D) GSVA results of GO terms (C) and KEGG pathways (D) based on the differentially expressed gene sets in the TCGA Seq dataset. [file Image_1.jpeg]

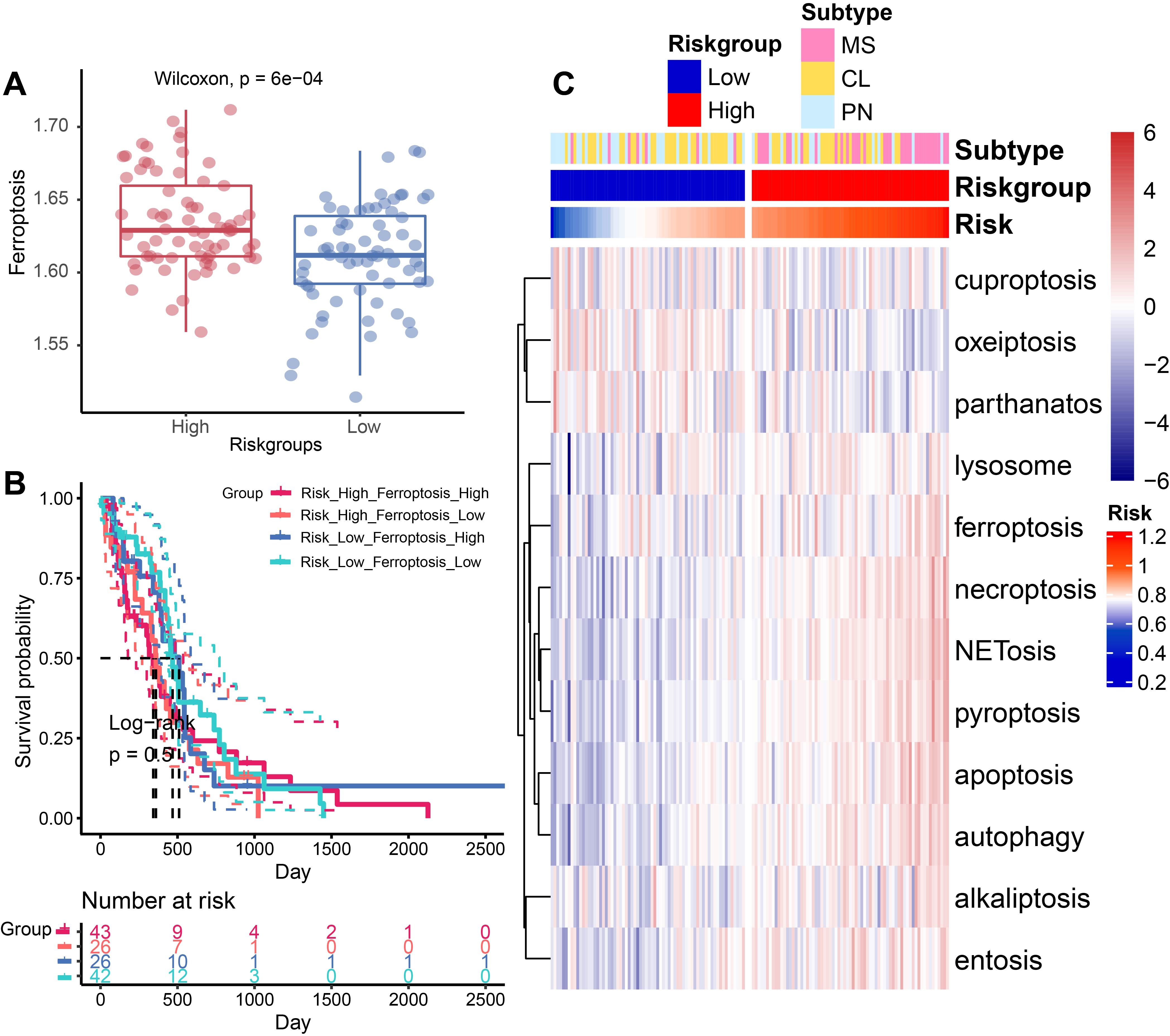

Supplement: Supplementary Figure 2 — Relationship between risk signature and different cell death modes in TCGA Seq dataset. (A) Ferroptosis regulators/markers were highly enriched in high-risk group based on the TCGA Seq dataset. (B) Survival analysis revealed no significance within the four groups on account of risk score and ferroptosis pathway level in the TCGA Seq dataset. (C) ssGSEA results of different cell death modes in TCGA Seq dataset. P < 0.05, statistically significant. [file Image_2.jpeg]

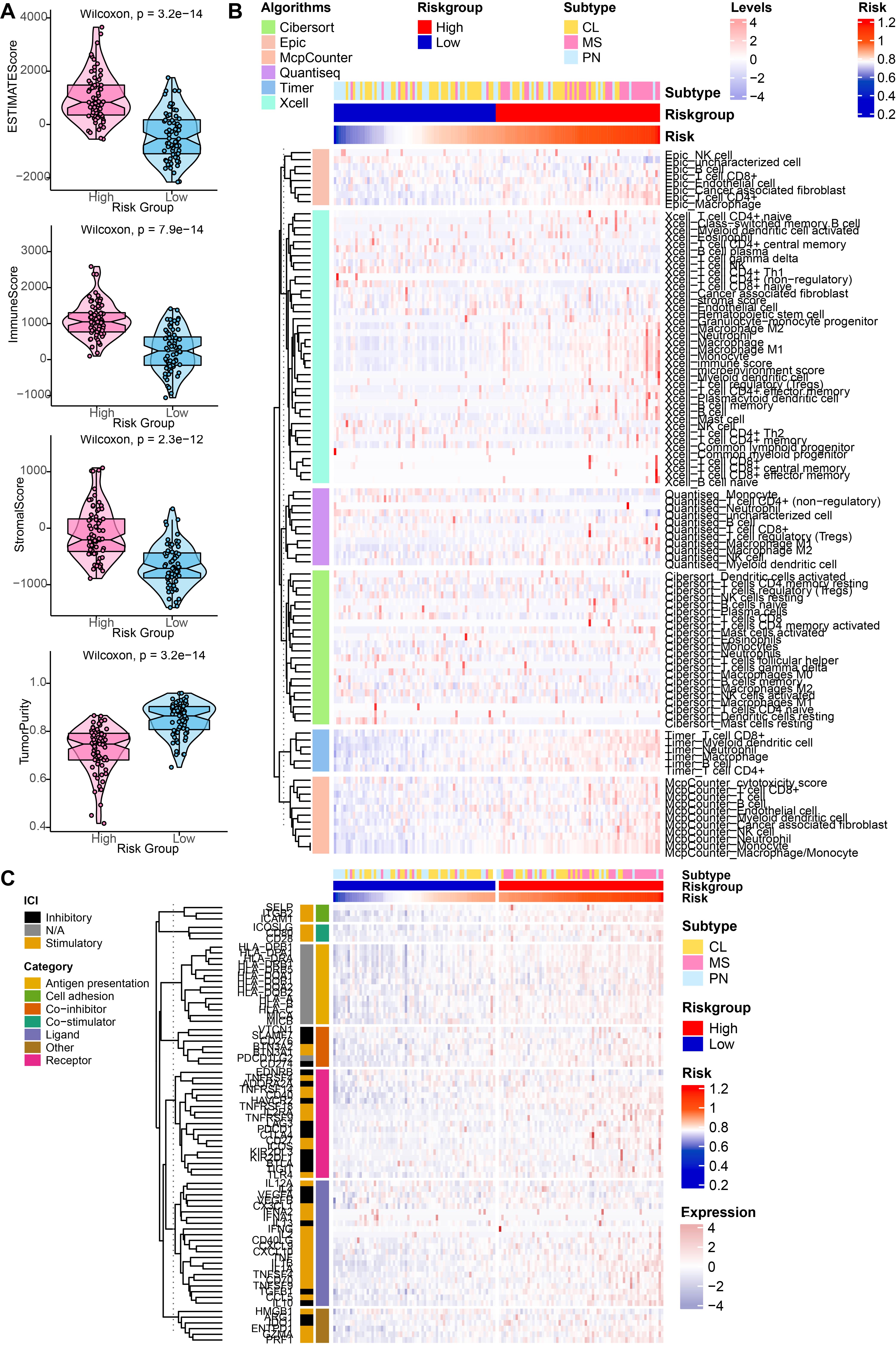

Supplement: Supplementary Figure 3 — Depiction of tumor immune microenvironment and immune associated genes in TCGA Seq dataset. (A) Calculation of the ESTIMATE score, ImmuneScore, StromalScore, and TumorPurity of the TCCA-seq samples. (B) Heatmap illustrated immune infiltration by performing the R package which integrates TIMER, MCP-counter, EPIC, xCell, quanTIseq, and CIBERSORT in the TCGA Seq dataset. (C) Heatmap revealed immune associated gene expression levels in the TCGA Seq dataset within the two risk groups. P < 0.05, statistically significant. [file Image_3.jpeg]

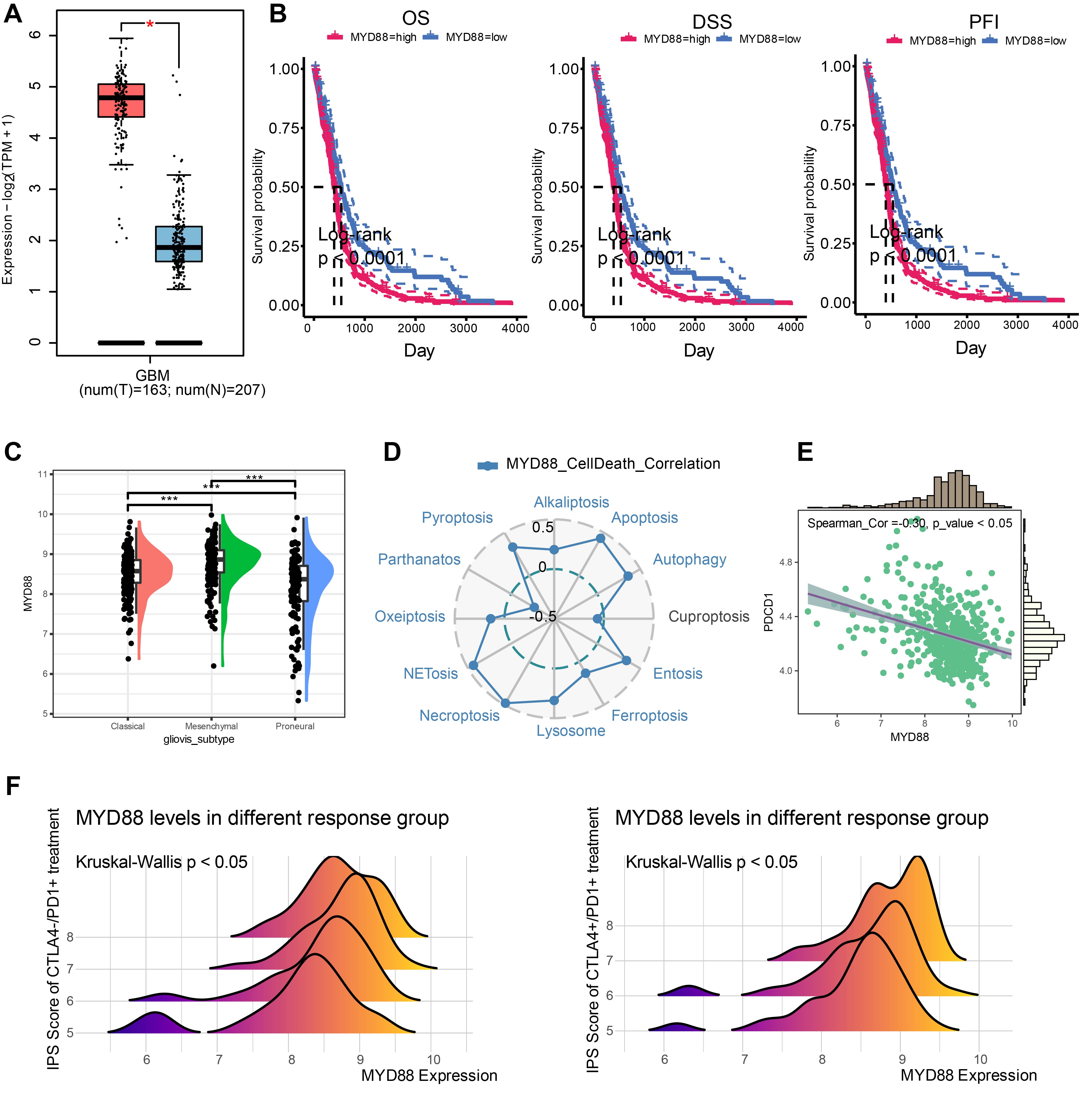

Supplement: Supplementary Figure 4 — ICD biomarker MYD88 was associated with clinical malignancy, cell death pathways, and IPS score. (A) Expression differences analysis of MYD88 in GBM and control tissue based on GEPIA2. (B) Survival analysis indicated that high MYD88 was associated with a dismal prognosis. (C) MYD88 expression differences in different GBM subtypes. (D) The correlation analysis indicated that MYD88 expression was associated with different kinds of cell death modes. (E) The correlation analysis revealed that MYD88 expression was negatively correlated with tumor PD-1 expression. (F) The MYD88 expression levels in GBM patients with different IPS scores. [file Image_4.jpg]
